# Cryotherapy: biochemical alterations involved in reduction of damage induced by exhaustive exercise

**DOI:** 10.1590/1414-431X20187702

**Published:** 2018-10-04

**Authors:** A.B.V. Furtado, D.D. Hartmann, R.P. Martins, P.C. Rosa, I.K. da Silva, B.S.L. Duarte, L.U. Signori, F.A.A. Soares, G.O. Puntel

**Affiliations:** 1 Centro de Ciências da Saúde, Programa de Pós-graduação em Reabilitação Funcional, Universidade Federal de Santa Maria, Santa Maria, RS, Brasil; 2 Centro de Ciências Naturais e Exatas, Programa de Pós-Graduação em Ciências Biológicas, Bioquímica Toxicológica, Universidade Federal de Santa Maria, Santa Maria, RS, Brasil; 3 Centro de Ciências da Saúde, Departamento de Morfologia, Universidade Federal de Santa Maria, Santa Maria, RS, Brasil

**Keywords:** Exercise-induced damage, Muscular damage, Reactive species, Cold-water immersion, Therapeutic cold

## Abstract

When exercises are done in intense or exhaustive modes, several acute biochemical mechanisms are triggered. The use of cryotherapy as cold-water immersion is largely used to accelerate the process of muscular recovery based on its anti-inflammatory and analgesic properties. The present study aimed to study the biochemical effects of cold-water immersion treatment in mice submitted to exercise-induced exhaustion. Swiss albino mice were divided into 4 treatment groups: control, cold-water immersion (CWI), swimming exhaustive protocol (SEP), and SEP+CWI. Treatment groups were subdivided into times of analysis: 0, 1, 3, and 5 days. Exhaustion groups were submitted to one SEP session, and the CWI groups submitted to one immersion session (12 min at 12°C) every 24 h. Reactive species production, inflammatory, cell viability, and antioxidant status were assessed. The SEP+CWI group showed a decrease in inflammatory damage biomarkers, and reactive species production, and presented increased cell viability compared to the SEP group. Furthermore, CWI increased acetylcholinesterase activity in the first two sessions. The present study showed that CWI was an effective treatment after exercise-induced muscle damage. It enhanced anti-inflammatory response, decreased reactive species production, increased cell viability, and promoted redox balance, which could decrease the time for the recovery process.

## Introduction

Chronic adaptations generated by regular physical exercise are well known for their capability to improve health and quality of life. In the long-term perspective, physical exercise causes regulation of metabolism and antioxidant status (1). However, when exercises are done in intense or exhaustive modes, several acute effects are triggered, including excessive inflammation, hormonal changes, and high production of reactive oxygen species, which may lead to oxidative stress, tissue damage, protein oxidation, lipid peroxidation, and DNA damage (1,2). These imbalances in the oxidative process during muscle contraction can contribute to decreases in contractile force, leading to exercise-induced exhaustion and consequently increased susceptibility to muscle damage (3).

It is well known that exercise-induced muscle damage generates an inflammatory response, which is followed by the muscle recovery phase (4). There are numerous methods used by sports medicine that aim to accelerate the muscle recovery process, such as cryotherapy (5). Although it is frequently used as a recovery method, controversies remain around the real benefits of this treatment. Previous researchers have shown that individuals submitted to cryotherapy have higher levels of antioxidants (6), decrease in oxidative stress (7), and lower levels of inflammation biomarker levels and mitochondrial dysfunction (8). On the other hand, other studies have proven that cryotherapy is not effective in muscle damage or inflammation biomarkers (9), or in decreasing oxidative stress induced by lesions (10).

One of the most popular methods of cryotherapy is cold-water immersion (CWI), which is known for its anti-inflammatory and analgesic effects obtained through extreme or moderate exposure of body segments in water below 15°C (11). Additionally, recent studies have shown that CWI is more efficient than other forms of recovery because it causes local vasoconstriction that leads to the reduction of fluid propagation in the interstitial space. Hence, this method favors the reduction of muscle damage, acute inflammation (12), muscle tissue temperature, venous O_2_ saturation, plasma myoglobin concentration, and swelling (13).

Despite extensive research on CWI, the results are still controversial, which is explained by the diversity of the protocols used in research (5,11,14,15). Such diversity promotes different physiological and biochemical mechanisms triggered by CWI that are not clearly described. In view of this, further studies are important, and then evidence-based guidelines can be developed (6). In a preview study by our group that evaluated the use of cryotherapy in muscle contusion, the use of low temperatures modulated biochemical response (7). Now, we tested a different protocol of muscle lesions induced by exercise and cryotherapy, used here as a continuous treatment with cold-water immersion to test the effect in most of the biochemical markers tested in our first study. Our swimming exhaustive protocol was designed to induce similar biochemical alterations as a skeletal injury model and the biochemical markers were selected taking into account this context. Considering this, the aim of this study was to establish which biochemical changes are induced by CWI treatment in mice submitted to exercise-induced exhaustion after muscle damage. Our hypothesis was that the CWI protocol would reduce the inflammatory process and reactive species formation, and would increase antioxidant status and cellular viability after exercise-induced muscle damage compared to passive recovery.

## Material and Methods

### Animals and reagents

Adult male Swiss albino mice weighing 30–50 g were used in this study. During the experimental protocol, animals were kept in cages of 10 animals each, with food and water *ad libitum*. Mice were maintained in a room with controlled temperature and a 12-h light/dark cycle. The room where the experimental procedures occurred had controlled temperature of 22°C±2. Assay reagents were purchased from Sigma (USA) and biochemical kits were obtained from the standard commercial supplier Labtest (Brazil). All the procedures were in accordance with the guidelines of the Committee on Care and Use of Experimental Animal Resources of the Universidade Federal de Santa Maria, Brazil (UFSM; #4185290915).

### Experimental groups

The animals (n=80) were randomized and divided into four homogeneous groups: 1) control: animals were not submitted to either protocol of muscle damage (swimming exhaustion protocol, SEP) or treatment (CWI); 2) CWI: animals were submitted only to the CWI protocol; 3) exhaustion: animals were submitted only to SEP; 4) SEP+CWI: animals were submitted to both protocols

All groups were subdivided into four different times of analysis: 0, 1, 3, and 5 days. The aim was to observe the evaluation of biomarkers in different periods after exhaustion protocol and single or repeated sessions of CWI treatment. The subgroup sample sizes were calculated by a power analysis based on Puntel et al. (8) and determined that four animals would provide a statistical power of 95% at an alpha level of 5%.

### Water adaptation

All animals were adapted to the water before the beginning of the experiment. The adaptation consisted of keeping the animals walking in shallow water at 31°C for 20 min for 7 days. The aim was to adapt the animals to the water environment without promoting physical training. Warm water was used because, as shown in our results, cold-water immersion could promote biochemical changes at the first contact.

### Swimming exhaustion protocol

The SEP and SEP+CWI groups were submitted to the SEP according to the method proposed by Huang (16) with some modifications. It consisted of a swimming exercise in a tank with controlled temperature (31°C) carrying constant loads of 10% of the body weight (17) that were fixed on mice tails. Exhaustion was characterized by the animal losing the coordinated movements and not returning to the surface within 7 s. The animals were submitted to the exercise only once on day 0.

### Cold-water immersion

**Figure 1:**
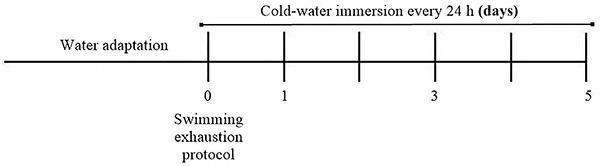
Timeline of the experiments.

After the exhaustion protocol, the animals of SEP+CWI were immediately put in a tank with controlled temperature (12°C) for 12 min following the protocol previously described by Machado et al. (15). The water depth was controlled so that the animal had the whole body submerged (except the head) without needing to swim to stay on the surface, that is, with the four paws resting on the floor of the tank. The animals of the CWI group were submitted to the same protocol. Both groups repeated the protocol every 24 h for 5 days (Figure 1). To minimize significant variations in the time scale of the endogenous temperature, after the cold-water immersion the animals were dried with towels and then re-allocated in the cages. This procedure was done at room temperature in order not to interfere with the physiological response to immersion.

### Tissue sampling

The animals were euthanized and blood was collected by heart puncture in previously heparinized syringe. Both gastrocnemius muscles were quickly removed, weighed, and placed on ice. Skeletal muscle tissue samples were homogenized within 10 min in 10 volumes of cold Tris 10 mM (pH 7.4) and centrifuged at 4000 *g* for 10 min at 4°C to yield the low-speed supernatant fraction (S1) that was used for different biochemical assays in all trials. Whole blood samples were centrifuged at 1500 *g* for 10 min at 4°C for plasma separation for biochemical analyses.

### Damage markers in skeletal muscle and plasma

#### Oxidized dichlorofluoresceine reactive species (DCF-RS) levels

DCF-RS levels were measured according to Perez-Severiano (19) with some modifications. Aliquots of skeletal muscle homogenate (50 μL) were added to a medium containing Tris-HCl buffer (10 mM; pH 7.4) and dichloro-dihydro-fluorescein diacetate (1 μM). The medium was then incubated in the dark for 1h until fluorescence measurement (excitation at 488nm and emission at 525 nm; both slit widths used were at 1.5 nm). DCF-RS levels were determined using a standard curve of DCF and the results were corrected by mg of protein.

#### Acetylcholinesterase (AChE) activity

AChE activity was estimated in skeletal muscle by the Ellman method (21) using a plate containing acetylcholine iodide (ATC) used as substrate and etopropazine as butyrylcholinesterase (BChE) inhibitor. Data were corrected by protein content and are reported in µmol of ATC hydrolyzed·min^−1^·mL^−1^.

*Creatine kinase (CK).* CK activity was measured spectrophotometrically in plasma samples by standard biological kits (Labtest, Brazil).

### Cell viability and antioxidant markers in skeletal muscle

#### Measurement of methyltetrazolium (MTT) reduction levels

Aliquots of skeletal muscle homogenate (90 µL) were added to a medium containing 1 mg/mL of MTT and were incubated in the dark for 60 min at 37°C. Then, 900 µL of DMSO was added. Formazan levels were measured spectrophotometrically at 570 nm and 630 nm and results were corrected by the protein content as proposed by Mosmann (18).

#### Non-protein thiol (–SH) levels

Non-protein –SH levels were determined according to the method proposed by Ellman (20) with some modifications. Samples of skeletal muscle homogenate (500 μL) were precipitated with 5% trichloroacetic acid (250 μL) and subsequently centrifuged at 1800 *g* for 10 min at 4°C.

The supernatant fraction (300 μL) was then added to a reaction medium containing TFK (0.5 mM, pH 7) and DTNB (20 mM). Non-protein –SH levels were measured spectrophotometrically at 412 nm. Results were calculated in relation to a standard curve constructed with GSH at known concentrations and corrected by the protein content.

### Protein quantification

The protein content was estimated by the Bradford method (22 [Bibr B23]) using bovine serum albumin (BSA) as the standard.

### Statistical analysis

Graphpad Prism 6 (USA) was used for all analyses. Data are reported as means±SD and variations between interventions are reported as mean differences (MD) and 95% confidence intervals (95%CI). Significance was assessed by two-way analysis of variance (ANOVA) followed by Tukey's *post hoc* test. Statistical significance was set at P<0.05. The main effects (effect size) were tested to reveal the size of the effect and complement P value (21) and are presented only when interactions between SEP and SEP+CWI were significant.

## Results

### Time to exhaustion

The mean swimming time of the exhaustive protocol was 343.3 s (±121.1).

### Damage markers in skeletal muscle and plasma

**Figure 2:**
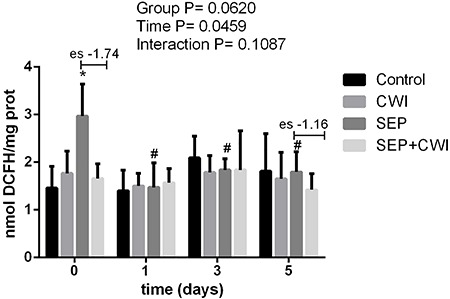
Effects of cold-water immersion (CWI) on dichloro-dihydro-fluorescein diacetate (DCFH) levels. Data are reported as means±SD (n=4). *P<0.05 for difference between groups at the same time-point; ^#^P<0.05 for difference between the group at different times (two-way ANOVA, followed by Tukey's *post hoc* test). es: effect size. SEP: swimming exhaustive protocol.

DCF-RS levels in skeletal muscle tissue are reported in Figure 2. SEP significantly increased DCF-RS levels immediately after the exercise (P<0.05) compared to control (MD –1.51 ηmol DCF/mg protein, 95%CI –2.446 to –0.58), CWI (MD1.20 ηmol DCF/mg protein, 95%CI 2.13 to 0.27), and SEP+CWI (MD 1.31 ηmol DCF/mg protein, 95%CI 0.38 to 2.24). For time analysis, there was a difference of the SEP group between day 0 and day 1 (MD 1.49 ηmol DCF/mg protein, 95%CI 0.53 to –2.46), day 3 (MD 1.12 ηmol DCF/mg protein, 95%CI 0.1662 to 2.089), and day 5 (MD 1.17 ηmol DCF/mg protein, 95%CI 0.2091 to 2.132). In effect size (es) analyses, there were differences between SEP and SEP+CWI groups on days 0 (es –1.74) and 5 (es –1.16).

**Figure 3:**
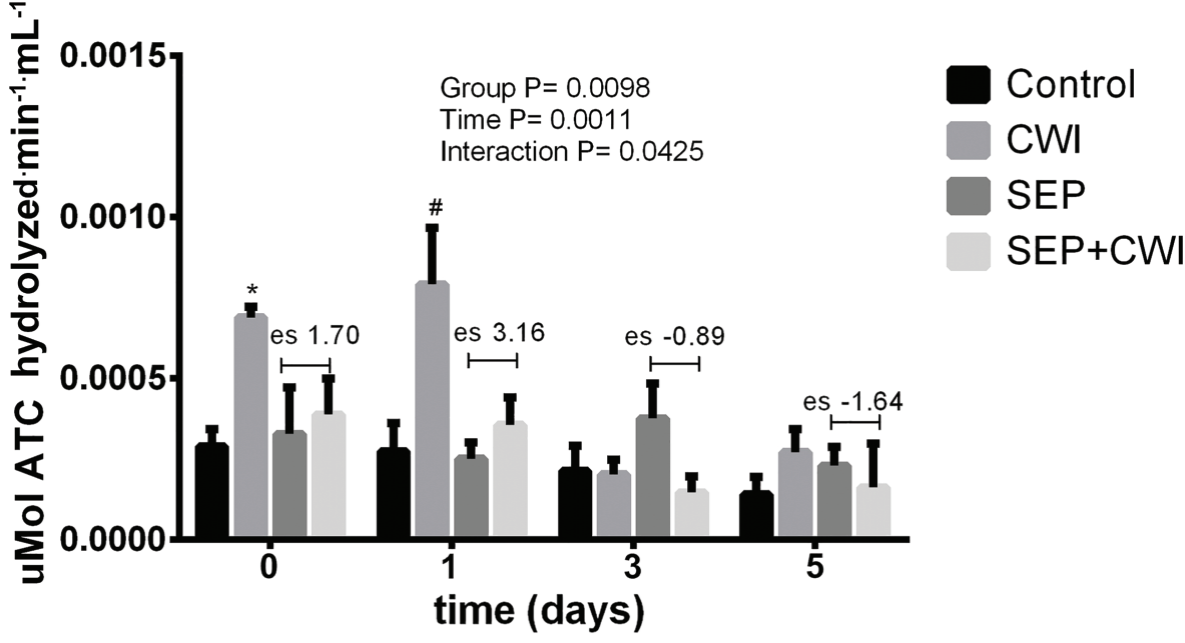
Effects of cold-water immersion (CWI) on acetylcholine iodide (ATC) activity. Data are reported as means±SD (n=4). *P<0.05 compared to control and swimming exhaustive protocol (SEP); ^#^P<0.05 compared to all other groups (two-way ANOVA, followed by Tukey's *post hoc* test). es: effect size

AChE activity in skeletal muscle tissue (Figure 3) increased in the CWI group only at day 0 (P<0.05) compared to the control group (MD –0.39×10^−3^ µmol ACT hydrolyzed·min^−1^·mL^−1^, 95% CI 0.7480×10^−3^ to –0.05×10^−8^.100^−5^) and the SEP group (MD 0.361×10^−3^ µmol ACT hydrolyzed·min^−1^·mL^−1^, 95% CI 1.25×10^−8^ to 0.70×10^−3^) and on day 1 (P<0.001) compared to all other groups (control, MD 0.0005188×10^−3^ µmol ACT hydrolyzed·min^−1^·mL^−1^, 95% CI 0.86 to 0.17; SEP, MD 0.54, 95%CI 0.19 to 0.88; SEP+CWI, MD 0.43×10^−3^ µmol ACT hydrolyzed·min^−1^·mL^−1^, 95%CI 8.7e-005 –0.78×10^−3^ µmol ACT hydrolyzed·min^−1^·mL^−1^). On time analysis, there were differences in CWI between days 0 and 3 (MD 0.0004859, 95%CI 0.0001362–0.0008355), 0 and 5 (MD 0.000417, 95%CI 6731e-005–0.0007667), 1 and 3 (MD 0.0005891, 95%CI 0.0002394–0.0009388), 1 and 5 (MD 0.0005203, 95%CI 0.0001706–0.0008699). In effect size analyses, there were differences between SEP and SEP+CWI groups on all days, however, at immediate time and on day 1, AChE increased in the SEP+CWI group (es 1.70; 3.16), and on days 3 and 5 the opposite occurred, AChE was increased in the SEP group (es –0.89; –1.64).

**Figure 4:**
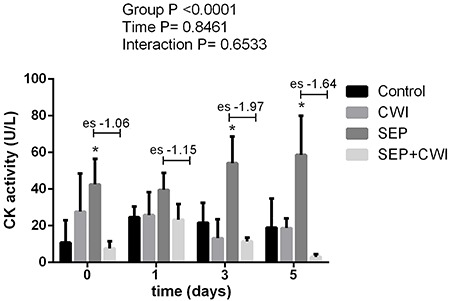
. Effects of cold-water immersion (CWI) on creatine kinase (CK) activity. Data are reported as means±SD (n=5). *P<0.05 compared to the other groups (two-way ANOVA, followed by Tukey's *post hoc* test). es: effect size; SEP: swimming exhaustive protocol.

In Figure 4, the positive effect of CWI on CK enzyme modulation can be observed in SEP+CWI. There was a difference between SEP and SEP+CWI groups on days 0 (P<0.05, MD 34,94 U/L, 95%CI 2.3 to 67.58), 3 (P<0.001, MD 42.64 U/L, 95%CI 9.995 to 75.28), and 5 (P<0.001, MD 55.68 U/L, 95%CI 23.04 to 88). These results showed an increase in the CK activity in the SEP group. In effect size analyses, the effects caused by CWI can be observed on days 0 (es –1.06), 1 (es –1.15), 3 (es –1.97), and 5 (es –1.64).

### Cell viability and antioxidant markers in skeletal muscle

**Figure 5:**
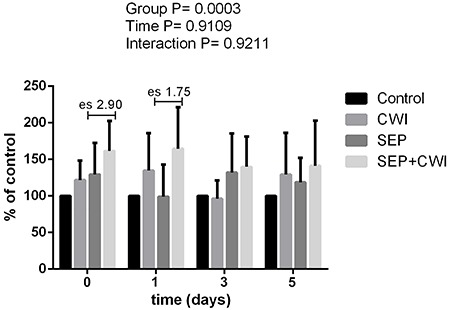
Effects of cold-water immersion (CWI) on MTT levels. Data are reported as means±SD (n=4). P>0.05 (two-way ANOVA, followed by Tukey's *post hoc* test). es: effect size; SEP: swimming exhaustive protocol.

After 5 days of CWI, the MTT levels (Figure 5) presented a significant value on group analysis (P=0.003, F(3,12)=14.31) but not on time analyses (P=0.911). Nevertheless, no significant interaction (P=0.921) between the groups was found. In the effect size analyses, MTT levels were increased in the SEP+CWI group on days 0 and 1 (es 2.90; 1.75).

**Figure 6:**
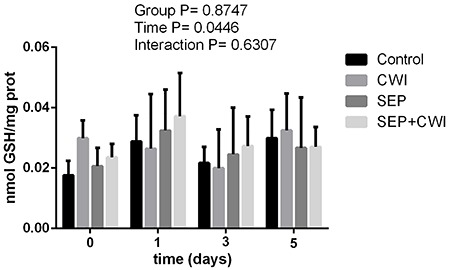
Effects of cold-water immersion (CWI) on non-protein –SH levels. Data are reported as means±SD (n=4). P>0.05 (two-way ANOVA, followed by Tukey's *post hoc* test). SEP: swimming exhaustive protocol.

Non-protein –SH levels (Figure 6) presented a significant value on time (P=0.0446, F(3,36)=2.970), but not on group analyses (0.8747) or interaction (0.6307). When Tukey's *post hoc* test was done, no significant difference was found.

## Discussion

In the current study, biochemical changes induced by CWI treatment in mice submitted to the SEP protocol were evaluated. Our study is the first to demonstrate an increase in ACHe activity in the first two sessions of CWI treatment and a decrease afterwards, indicating a modulation of the inflammation system. To the best of our knowledge, this is the first study to demonstrate the use of CWI for five days consecutively after a single exhaustive exercise session, which depicts a significant alteration in muscle recovery.

The most popular reason for using CWI is that it induces vasoconstriction, which leads to venous return increase, metabolite removal (24), and decreased muscle oxygen saturation (14), restricting the infiltration of inflammatory cells into the muscle (25). These mechanisms protect the uninjured tissue from enzymatic reactions triggered by exercise-induced damage (26). The proposed exhaustive protocol was able to evoke alterations in biochemical markers that clearly indicate muscle damage, such as increased CK activity and DCF-RS production, although the protocol did not lead to excessive damage, which may be equivalent to real sportive situations. Our data demonstrated that CWI treatment after an intense exercise bout was capable of decreasing reactive oxygen species (ROS) formation and damage, as well as increasing cellular viability. The results supported the hypothesis that CWI is better than passive recovery after exhaustive exercise and corroborate other studies that suggest that CWI treatment is an effective intervention after exhaustive exercise (5).

There are multiple mechanisms involved in oxidant/antioxidant status during exercise (27). Excessive levels of ROS may alter the muscle, mainly through inflammatory processes (28). Levels of DCF-RS were increased in the SEP group; however, the SEP+CWI group did not present an increment, which demonstrated that CWI may prevent the DCF-RS increase caused in our protocol of exercise-induced exhaustion. This finding clearly demonstrated that CWI was effective in limiting RS production just after exercise.

Considering that acetylcholine (ACh) is well known to inhibit tumor necrosis factor-α, interleukin-1β, and macrophage migration (29), the increased AChE activity may indicate a modulation in ACh levels and in its ability in regulating inflammatory processes (30). In the present study, AChE activity was increased in the CWI group. This unexpected result may be explained by the thermodynamic characteristics of AChE. Klichkhanov and Meilanov (31) reported that when rat erythrocyte membranes are exposed to hypothermia, there is an increase in the degree of substrate inhibition for AChE to the maximum rate and in the Michaelis Menten constant. On the other hand, when exposed to situations of hyperthermia, such as 42–48°C, 60% of AChE is inactivated (32). Regarding thermodynamics, AChE was increased in CWI, although not in the same magnitude as the exercise situations (SEP and SEP+CWI groups), as a result of exercise-induced hyperthermia. A possible explanation is that cold promoted isometric contractions as an adaption strategy in the first two applications of CWI. After that, the animals were adapted, and therefore, these contractions and AChE activity decreased. Since the increase was observed in the groups that were only submitted to CWI, this finding leads us to believe that exhaustive exercise partially inhibited adaptive effects. Furthermore, regarding only the SEP and SEP+CWI groups, CWI decreased AChE activity 24 h after damage, which showed that the inflammatory process was likely decreased.

It has already been shown that cryotherapy can weaken or delay the infiltration of inflammatory cells (8,33), which could be explained by vasoconstriction caused by exposure to the cold. This causes the reductionof cellular permeability, and lymphatic and capillary vessels, and consequently reduces fluid propagation into the interstitial space (34). In our study, CK levels increased on all days in the SEP group; however, the same pattern was not observed in the SEP+CWI group. This indicated that SEP was able to cause some degree of muscle damage, which was limited by the CWI protocol used here. Furthermore, the difference between treated and non-treated groups remained for 24–48 h, which is the peak of inflammatory process (35). This result demonstrated that CWI may be an effective intervention after exercise-induced damage even in the most acute phases of inflammation.

Mitochondria plays a key role in energy supply and operates as an indicator of cell viability (36). MTT reduction depends on the oxidoreductase enzyme family activity, such as dehydrogenase enzymes, and it is mainly located in mitochondria (37). In light of this, MTT reduction assay can be used as an indicator of cell viability. In our experiments, CWI improved cell viability through the increase of MTT reduction levels immediately and during the first 24 h. This result proved that CWI treatment might act by preserving skeletal muscle cell structure and improving cell viability even after exhaustive exercise. Our findings indicated that skeletal muscle cells could have a faster recovery from any damage suffered during exercise once cell viability is preserved by CWI.

In summary, we demonstrated from a biochemical perspective that CWI is an effective option of treatment after exercise-induced muscle damage. CWI modulated anti-inflammatory response, decreased reactive species production, increased cell viability, and promoted redox balance, which produced an improvement in the recovery process compared to a passive recovery. This reduction in muscle damage promoted by CWI is relevant and might be used as a basis for future research and in evidence-based clinical practice. The comparison of methodologies in future studies is necessary to conclude which are the most effective.
